# Prevalence of probable mental, neurological and substance use conditions and case detection at primary healthcare facilities across three districts in Ghana: findings from a cross-sectional health facility survey

**DOI:** 10.1186/s12888-023-04775-z

**Published:** 2023-04-20

**Authors:** Kenneth Ayuurebobi Ae-Ngibise, Lionel Sakyi, Lyla Adwan-Kamara, Crick Lund, Benedict Weobong

**Affiliations:** 1 Ghana Somubi Dwumadie (Ghana Participation Programme), PMB 6 Asoyi Crescent, East Legon, Accra, Ghana; 2 Kintampo Health Research Centre, Research and Development Division, Ghana Health Service, Kintampo North Municipality, Bono East Region, Ghana; 3 Centre for Global Mental Health, Health Service and Population Research Department, Institute of Psychiatry, Psychology and Neuroscience, King’s Global Health Institute, King’s College London, London, UK; 4 Department of Psychiatry and Mental Health, Alan J Flisher Centre for Public Mental Health, University of Cape Town, Cape Town, South Africa; 5 Department of Social and Behavioural Sciences, College of Health Sciences, University of Ghana, Accra, Ghana

**Keywords:** Mental health conditions, Detection rates, Prevalence, Primary healthcare

## Abstract

**Background:**

Few studies have examined the prevalence of mental, neurological and substance use (MNS) conditions, case detection and treatment in primary healthcare in rural settings in Africa. We assessed prevalence and case detection at primary healthcare facilities in low-resource rural settings in Ghana.

**Methods:**

A cross-sectional survey was conducted at the health facility level in three demonstration districts situated in Bongo (Upper East Region), Asunafo North (Ahafo Region) and Anloga (Volta Region) in Ghana. The study participants were resident adult (> 17 years) out-patients seeking healthcare at primary care facilities in each of the three demonstration districts. Data were collected on five priority MNS conditions: depression, psychosis, suicidal behaviour, epilepsy and alcohol use disorders.

**Results:**

Nine hundred and nine (909) people participated in the survey. The prevalence of probable depression was 15.6% (142/909), probable psychotic symptoms was 12% (109/909), probable suicidal behaviour was 11.8% (107/909), probable epilepsy was 13.1% (119/909) and probable alcohol use disorders was 7.8% (71/909). The proportion of missed detection for cases of depression, self-reported psychotic symptoms, epilepsy and alcohol use disorders (AUD) ranged from 94.4 to 99.2%, and was similar across study districts. Depression was associated with self-reported psychotic symptoms (RR: 1.68; 95% CI: 1.12–1.54). For self-reported psychotic symptoms, a reduced risk was noted for being married (RR: 0.62; 95% CI: 0.39–0.98) and having a tertiary level education (RR: 0.12; 95% CI: 0.02–0.84). Increased risk of suicidal behaviour was observed for those attending a health facility in Asunafo (RR: 2.31; 95% CI: 1.27–4.19) and Anloga districts (RR: 3.32; 95% CI: 1.93–5.71). Age group of 35 to 44 years (RR: 0.43; 95% CI: 0.20–0.90) was associated with reduced risk of epilepsy. Being female (RR: 0.19; 95% CI: 0.12–0.31) and having a tertiary education (RR: 0.27; 95% CI: 0.08–0.92) were associated with reduced risk of AUD.

**Conclusions:**

Our study found a relatively high prevalence of probable MNS conditions, and very low detection and treatment rates in rural primary care settings in Ghana. There is a need to improve the capacity of primary care health workers to detect and manage MNS conditions.

## Background

An estimated 10% of Ghanaians have common mental health conditions, while 1–3% have severe mental health conditions such as schizophrenia [1, 2]. Yet only 2% of such persons will receive treatment [3]. Epilepsy for instance is a common neurological disorder especially in poor areas of the world, the burden of it in low andmiddle-income countries LMICs is more than twice that found in high-income countries [4]. In Ghana epilepsy is the most common treated disorder among adolescents receiving services in four health facilities in the two districts in the Bono East Region [5].

As in many other low and middle-income countries (LMICs), few data are available in Ghana to inform the scale up of mental health services and reduce the large treatment gap [6, 7]. There is little epidemiological data on persons living with mental health conditions who attend primary care settings in Ghana, and to the best of our knowledge, none in the northern and central parts of the country [8]. In LMICs, lack of reliable data on mental health systems affects mental healthcare delivery efforts as more than one-quarter of these countries have no system for reporting basic mental health information [9]. In addition, empirical research is seldom used to guide policy development and implementation, especially in LMICs [10, 11]. Out of 136 global studies of scaling up mental health programmes according to a systematic review, only 15 were carried out in LMICs [12].

It is important to conduct studies of prevalence and needs for care in primary care settings in remote rural areas of Ghana for two main reasons: first to assess the need; and second to provide baseline data to assess the impact of proposed programmes to integrate mental health into primary care and narrow the treatment gap. In Ghana, notwithstanding the existence of the Mental Health Act (Act 846) since its passing in 2012 [13, 14], the infrastructure and public services have not been properly developed, including mental healthcare services to align with the population growth [15, 16]. For example whilst integration of mental health services in primary healthcare is a globally accepted approach to optimising healthcare in view of its capacity to meet multiple health and social needs from a single platform of care [17], mental health services are not integrated in primary healthcare in Ghana due to lack of resources and prioritisation [13, 18].

The WHO, in an effort to reduce this mental health treatment gap, has made significant efforts to build capacity for mental health services in LMICs through the introduction of the Mental Health Gap Action Programme (mhGAP) [6, 19]. The mhGAP provides key strategies such as integrated management plans and evidence-based guidelines for scaling up mental health services in LMICs for priority mental health conditions including depression, psychosis, alcohol use disorder, and epilepsy [20]. In the light of these global efforts at improving mental health services in LMICs, Ghana Somubi Dwumadie (Ghana Participation Programme), a four-year disability programme, is supporting the generation of evidence to inform the scale-up of quality integrated mental healthcare in primary care in Ghana. Part of the programme is to support selected districts to develop and implement mental healthcare plans including the capacity development of primary healthcare workers through mhGAP training. The implementation and evaluation of mental health programmes in real-world primary care and community settings is critical to identify factors that contribute to the effective scale-up of mental health services [21]. However, very few high-quality evaluations have been carried out in LMICs [7, 22], where scaling up mental health services is of great importance. Evaluation studies done in LMICs have not assessed important baseline factors such as case detection within routine primary healthcare facilities [21].

Ghana Somubi Dwumadie, together with the Ghana Health Service and the Mental Health Authority in Ghana is mindful of these limitations and is supporting an approach that can inform the systematic scale-up of integrated mental health services. This is being done through demonstration sites in three districts of Ghana. The goal of this paper is to report on prevalence and mental health case detection in three districts in three regions in order to provide a baseline estimate for assessing the impact of subsequent district mental healthcare plans.

## Methods

### Study setting

**Fig. 1:**
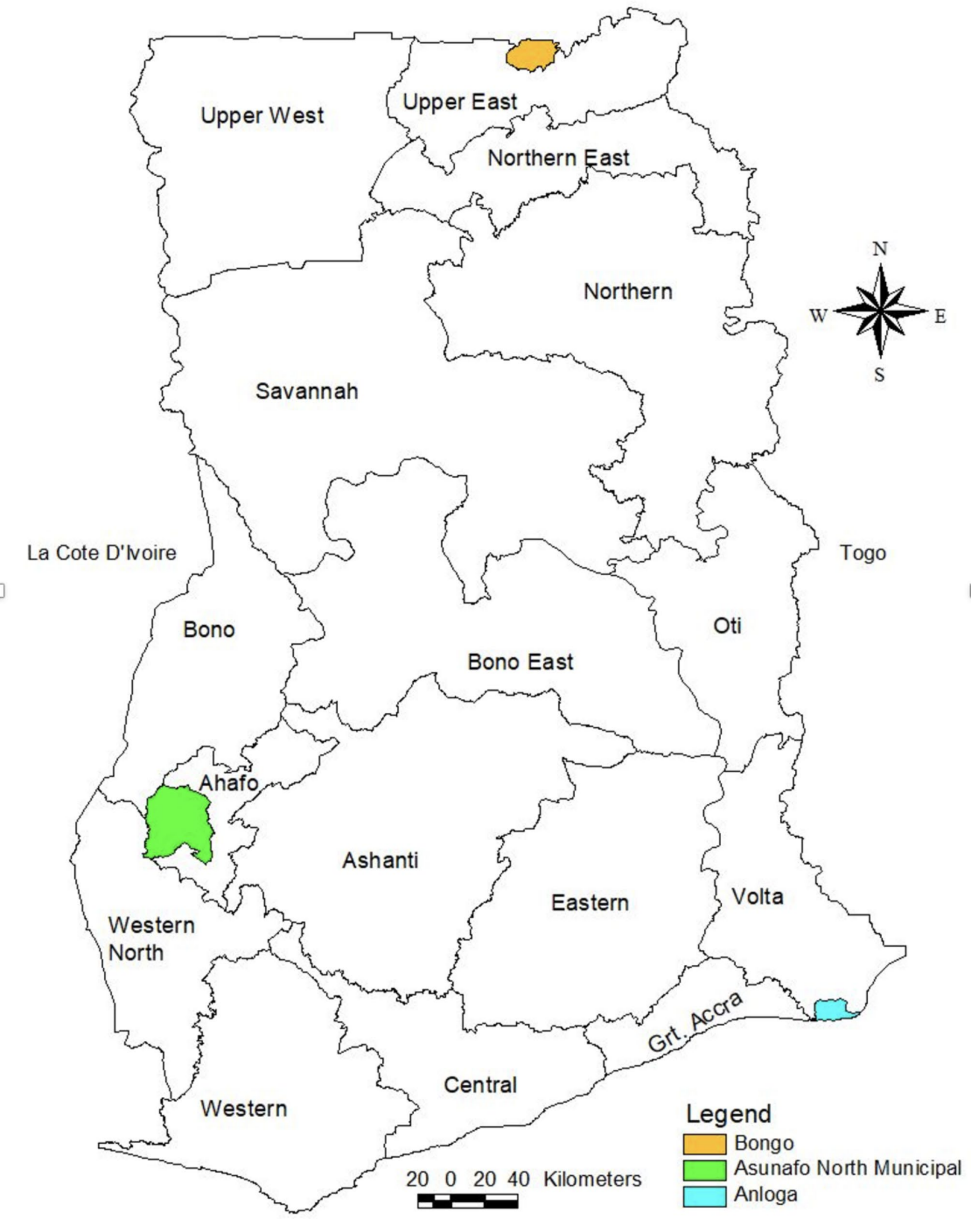
Map of Ghana showing the three demonstration districts

The study was conducted in 15 primary healthcare facilities across three districts purposively selected (out of 216 districts in Ghana) as demonstration sites for implementation of district mental healthcare plans. These plans include detailed implementation strategies to achieve integrated mental healthcare in primary healthcare settings, as part of Ghana Somubi Dwumadie [23]. These districts included Anloga (population: 99,996) in the Volta Region (Southern belt), Asunafo North (population: 157,732) in the Ahafo Region (Middle belt) and Bongo (population: 105,206) in the Upper East Region (Savannah belt) (Fig. 1: Map of Ghana showing the three demonstration districts). The selection of the ‘demonstration’ districts was guided by a set of criteria outlined in Ghana Somubi Dwumadie’s framework for implementing district mental healthcare plans, ratified in 2020 in a high-level stakeholder engagement meeting in Accra, Ghana [24]. The criteria for selection included: geo-political equity (each of the districts with health facilities should spread across the three zones of Ghana: Southern, Middle Belt, and Savannah); representativeness (i.e. the districts should not be over-resourced, but also not under-resourced, particularly in relation to human resources so that lessons could be generalised to inform scale up in other districts); an appropriate level of prevailing mental health activity (i.e. there should be no existing sites for mental health research or previous/ or on-going district mental health plans or national academic centres for mental health should exist as this would create an unrealistic environment which is not representative of other districts); and willingness of district leadership to engage. Two key stakeholders that were involved in selecting the districts included Ghana Health Service (GHS) and the Mental Health Authority (MHA).

The 15 health facilities are presented on Table 1. These included 10 health centres, 2 hospitals, 2 Community Health Planning and Services and 1 clinic.

**Table 1:** List of Health Facilities in 3 demonstration districts, 2019–2021

District	Facility name	OPD Attendance			Facility Type	Ownership
		2019	2020	2021		
Anloga	Tegbi	12,027	11,011	NA	Health Centre	Public
	Dziedzorve	3438	3140	NA	CHPS	Public
	Kotoka Memorial	2446	1392	NA	Health Centre	Public
	Tregui	1361	951	NA	Health Centre	Public
	Anloga	10,035	9955	NA	Health Centre	Public
Bongo	Namoo	9287	5504	4479	Health Centre	Public
	Dua	5775	4219	2926	Health Centre	Public
	Kodorogo	977	853	345	Health Centre	Public
	Bongo	23,102	21,481	18,002	Hospital	Public
	Wagliga	4133	2 814	2240	CHPS	Public
Asunafo North	Akrodie	NA	30,958	18,274	Health Centre	Public
	Mim	NA	19,512	11,194	Health Centre	Public
	Kasapine	NA	4486	3158	Clinic	Public
	Asumura	NA	1780	4325	Health Centre	Public
	Goaso	NA	55,446	44,010	Hospital	Public

*NA: information on OPD attendance not available

*CHPS: *Community Health Planning and Services*

Following the selection of districts, primary healthcare facilities were identified for the baseline facility survey. Within each district five health facilities were randomly selected from a pool of 6–10 health facilities with high OPD attendance, including the availability of physician assistants, mental health nurses or midwives. On the average these health facilities had a minimum of 48 OPD daily attendance visits.

### Study design and research participants

We employed a cross-sectional health facility detection survey design. The baseline survey (the focus of this paper) was conducted from November 2021 to December 2021, prior to the implementation of the district mental healthcare plans and mhGAP in each demonstration district. We estimated a minimum sample size of 240 participants in each district to detect a 15% increase in case detection at 80% power, 5% alpha and a design effect of 2. This is based on the test of our primary hypothesis that there will be no difference in case detection and initiation of treatment (defined as proportion of patients with the target MNS conditions receiving diagnosis and initiation of treatment) between baseline and endline (after primary health care worker training).

Data were collected on five priority mental, neurological and substance use (MNS) conditions of interest in the mhGAP-IG namely: depression, psychosis, suicidal behaviour, epilepsy and alcohol use disorders (AUD) to assess the prevalence and detection of MNS conditions at the routine primary healthcare facilities. Each data collector was assigned to a health facility for the daily data collection for the period used for the data collection.

Eligible participants were resident adult (> 17 years) out-patients seeking healthcare at primary care facilities in each of the three demonstration districts. All consecutive out-patients exiting their clinical consultations were approached and those who consented to participate were recruited. Nine trained and experienced data collectors administered a two-part survey adapted from the Programme for Improving Mental health care (PRIME) in five LMICs [21]: first, a battery of five screening measures to elicit responses on symptoms and help-seeking behaviour for depression, psychosis, suicidal behaviours, epilepsy, and AUD; and second, a quantitative/qualitative exit interview to collect data on the patient’s experience regarding the clinical consultation. Each interview session lasted between 30 and 40 min and we did not encounter patients that refused consent to participate in the study. This section of the form also included extraction of data on clinical notes from individual patient folders.

### Measures

The measures included items on participant socio-demographic identifiers and a battery of instruments including: the Patient Health Questionnaire (PHQ-9), Psychosis screener, Suicidal Ideation screener, Epilepsy Screener, and Alcohol Use Disorders Identification Test. Some of the measures (suicidal ideation screener, epilepsy screener) were from the PRIME study. The other measures have been previously used in Ghana [25, 26]. Training on the English version of the measures was conducted by BW, AK, and LS. Experienced bi-lingual (English and the predominant local language in the study districts) data collectors were trained in a 3-day workshop on the content and administration of all study questionnaires, including the exit interview. This involved forward translation to the local languages (Gurene, Twi/Bono, and Ewe), and back translation, particularly paying attention to key constructs in the backtranslation. Consensus on the translation of each item was obtained. The data collectors were trained on how to administer the study tools. They were first introduced to mental health and the various conditions especially the priority MNS disorders. The trainees systematically went through all the questions in each study tool and clarified all concerns regarding how the questions should be asked and the expected responses. Interviewing skills was treated and a session on the translation of key constructs from English to the local languages. Both theory and practical sessions were employed. Trainees performed role plays for the facilitators to assess their competencies and understating of key constructs. There were also group work and mock exercises involving the translation and administering the questionnaires in the various local languages and documentation of key words in the local languages.

During the data collection, all study participants provided written consent before completing the questionnaire in the local language of their choice (Ewe, Gurene, and Twi), mostly based on the geographical location of the health facility although some interviews were also conducted in English. The trained data collectors administered the structured questionnaires in the participant’s primary language, using an electronic mobile device or tablet. All the questionnaires were administered by the data collectors. For the purposes of this study, these measures were used for diagnosis of the priority MNS conditions, and there was no further clinical assessment to validate the diagnosis of these screening questionnaires. The screening tools used for the data collection are reliable and valid instruments that have been used widely to screen for priority MNS conditions [27, 28]. Each measure is described in detail below.

#### Patient health questionnaire (PHQ-9)

The PHQ-9 is a structured questionnaire that enquires after the nine symptom-based criteria for a diagnosis of DSM-IV depression, including their duration and severity [29]. Each item is scored on a scale of 0 to 3 and generates a continuously distributed total score ranging from 0 to 27. In its initial review the PHQ-9 recorded sensitivity and specificity of 0.88 at a cut-off of 10 [29], and high positive predictive value [30]. The PHQ-9 has been previously validated in Ghana and showed superior psychometric properties when compared with the Edinburgh Postnatal Depression Scale [25, 26]; it recorded a sensitivity of 94% and specificity of 75% at a cut-off score of 5. The measure has subsequently been used in other studies [26, 31]. Probable depression ‘diagnosis’ (major or minor) is defined in this study as having occurred when a total score of at least 5 is indicated, and at least two cardinal symptoms of depression are reported as present for at least most of the time in the last two weeks; these must include depression or anhedonia (loss of interest or pleasure). In this study, the PHQ-9 recorded a Cronbach’s reliability coefficient of 0.83, indicating an acceptable level of reliability.

#### Psychosis screener

The psychosis screener used in this study (adapted from the PRIME study) is a structured 10-item questionnaire that enquires after symptoms of psychosis, which require a yes or no answer. Only items 1–6 are scored, and these include items such as strange feelings, having special powers, felt other people are too interested in you, felt thoughts were directly interfered with, ever heard voices or participant had ever been prescribed anti-psychotic medicine. The cut-off point applied for recording a person as screen positive for ‘probable psychotic symptoms’ was endorsing at least 2 items on the screener. In this study, this instrument recorded a Cronbach’s reliability coefficient of 0.7, which is an acceptable level of reliability.

#### Suicidal ideation and action measure

The suicidal ideation and action measure is a structured a 9-item questionnaire (adapted from PRIME) that enquires after signs and symptoms of suicidal thought and behaviour, including help-seeking. For this study we defined probable suicidal behaviour broadly as comprising of three domains (thoughts, plan, and attempt). Three items covering these domains that provide a ‘yes’ or ‘no’ answer were used; any ‘yes’ response was considered a screen positive for probable suicidal behaviour. In this study, using only three items it recorded a Cronbach’s reliability coefficient of 0.4, indicating a low reliability.

#### Epilepsy screener

The epilepsy screener is a structured 10-item measure (adapted from PRIME) with two sub-scales on symptoms and severity. The epilepsy screener seeks to establish if the patient has ever had a fit, or epilepsy. It assesses symptoms such as falling to the ground with loss of consciousness without reason and experiencing twitching, shaking of the arms of legs without control, wetting yourself or biting of the tongue. It further seeks to confirm if a clinician had ever confirmed an index person as having epilepsy, last time index person experienced epileptic attack and the number of seizures recorded in the last 30 days. The symptom sub-scale elicits ‘yes’ or ‘no’ responses. For this study, a probable ‘diagnosis’ of epilepsy entailed a ‘yes’ response to any of the 6 questions on the symptom sub-scale. In this study, it recorded a Cronbach’s reliability coefficient of 0.5, indicating a low reliability.

#### Alcohol Use Disorders Identification Test (AUDIT)

The AUDIT is a 10-item screening tool developed by the WHO for the detection of hazardous and harmful alcohol use including alcohol dependence in primary healthcare settings [32]. Each item is scored on a scale of 0 to 4 and generates a continuously distributed total score ranging from 0 to 40. In the initial validation involving 6 countries, scores of 8–15, 16–19, and 20 or more, represented probable diagnosis of hazardous use, harmful use, and alcohol dependence respectively [33–35]. The initial validation generated sensitivities around 0.90 and specificities averaging 0.80 [36]. In a recent review of the AUDIT in LMICs, lower cut-off scores were observed [37]: hazardous drinking cut-off scores of > 3 or > 5 yielded sensitivities ranging from 93.5 to 96.2%, and specificities of 63.3–91.5%; harmful drinking cut-off score of > 7 or > 8 yielded 90.0% sensitivity, 86.2% specificity; dependent drinking cut-off scores of > 7 to > 24, yielded sensitivity 63.6% and specificity of 75%. In this study, it recorded a Cronbach’s reliability coefficient of 0.86, which confirms the screening tool to have acceptable reliability. For this study a probable ‘diagnosis’ of any AUD was defined as a total score of 8 and above.

#### Consultation experience and folder data extraction

This section of the survey included both quantitative and qualitative questions on details of the consultation, and was used to ascertain detection of the priority MNS. The first part which was fully structured, extracted data from clinical folders of patients. The information included type of clinician seen, symptoms presented, diagnosis and treatment prescribed including any referrals made by the clinician.

The research team engaged the services of pharmacists at the district hospitals to support the data collectors with extraction of data on whether or not a diagnosis was given for each of the priority MNS conditions (except suicidal behaviour as this was assessed separately for those patients at risk and referral made where necessary). The support from the pharmacists was instructive in ensuring the diagnosis on patients folders are correctly captured, where the information was not clear to the data collectors. The second part was a self-report administered to the patients on whether or not the health worker made a diagnosis or mention if the patient had a health problem (e.g. Did the [health worker] give you a diagnosis of depression?). Given this was a self-report, only the folder-extracted data was used for this analysis. Other areas assessed included duration of clinical consultation, type of clinician seen, medication given and whether adequate advice was provided on how to take the medications. Participants that needed referral based on the screening were referred to the mental health unit for help.

### Data analysis

Both descriptive and inferential analyses were conducted by the investigators. Descriptive analyses were performed to estimate the prevalence of the probable diagnoses of the priority MNS conditions (assessed by the survey team) and the proportion detected by health care workers at the health facilities. Prevalence estimates were computed and reported with 95% CI. To estimate detection proportions, positive cases (i.e. met criteria for depression, psychosis, suicidal behaviour, epilepsy or AUD) screened by the survey team were compared with the health worker diagnosis. A priority MNS was classified as ‘missed detection’ if a solicited diagnosis was not provided in the patient folder by the health worker but was screened positive for any of the priority MNS conditions by the independent survey team using the study screening tools. Following this, inferential analyses were conducted to ascertain patient-level correlates of ‘missed detection’ and prevalence of the priority MNS conditions. For the analysis on correlates of ‘missed detection’, Fisher’s exact chi-square test (opted for because of insufficient cell observations (< 5)) was used to determine if there was a significant association between case detection (except suicidal behaviour as health worker data was not collected) and each measured patient level factor (age, sex, marital status, education, employment and district of residence); p-values were reported with statistically significant level determined at 0.05. For the analysis of correlates of the prevalence outcomes, logistic regression was used to examine associations first between potential determinants (patient level factors) and the prevalence of each of the priority MNS conditions. Second, associations between other priority MNS conditions and prevalence of depression (as the outcome) were examined. Effect sizes are reported as crude and adjusted relative risks (aRR) estimated using the marginal standardisation technique with 95% confidence intervals estimated via the delta method [38]. Analyses were conducted using Stata 14 [39], charts were generated using Microsoft Excel.

## Results

### Socio-demographic characteristics of study population

A total of 909 people (Bongo district: 301, Asunafo North district: 308, and Anloga district: 300) attending primary health facilities within the period (November – December 2021) participated in the study of which approximately 82% (741/909) were women, 30% (273/909) aged 18–24 (Table 2). Approximately 65% (587/909) of the participants were married, 29% (265/909) had no formal education, while 64% (583/909) had ever been employed.

**Table 2:** Baseline socio-demographic correlates of priority MNS among study participants across the demonstration sites

Correlates	Participants	Depression		Psychosis		Suicidal Behaviour		Epilepsy		AUD	
	n (%) of participants (N = 909)	n (%) with depression (N = 142)	RR_1_ (95% CI)	n (%) with psychosis (N = 109)	RR_1_ (95% CI)	n (%) with suicidal behaviour (N = 107)	RR_1_ (95% CI)	n (%) with epilepsy (N = 119)	RR_1_ (95% CI)	n (%) with AUD (N = 71)	RR_1_ (95% CI)
**Age Group**											
**18–24**	273(30.03)	37 (13.55)	1	36(13.19)	1	35(12.82)	**1**	43(15.75)	1	9(3.30)	1
**25–34**	250(27.50)	35(14.0)	1.010.62–1.63)	27(10.80)	0.67(0.41–1.11)	24(9.60)	0.77(0.45–1.32)	26(10.4)	0.68(0.41–1.12)	11(4.40)	1.17(0.46–2.99)
35–44	136(14.96)	28(20.59)	1.62(0.95–2.78)	17(12.50)	0.88(0.49–1.61)	18(13.24)	1.01(0.53–1.93)	10(7.35)	**0.43(0.20–0.90)**	16(11.76)	3.06(1.14–8.24)
**45–54**	129(14.19)	20(15.50)	1.36 (0.74–2.50)	11(8.50)	0.62(0.30–1.28)	16(12.4)	0.87(0.43–1.76)	16(12.4)	0.63(0.32–1.24)	16(12.4)	3.10(1.13–8.52)
**>/=55**	121(13.31)	22(18.18)	1.54(0.83–2.86)	18(14.80)	0.82(0.42–1.59)	14(11.57)	0.71(0.33–1.52)	24(19.83)	1.08(0.58–2.02)	19(15.7)	**3.00(1.07–8.43)**
**Sex**											
Male	168(18.48)	27 (16.07)	1	18 (10.71)	1	16(9.52)	1	27(16.07)	1	36(21.4)	1
Female	741(81.52)	115 (15.52)	1.01 (0.67–1.53)	91 (12.28)	0.98(0.62–1.57)	91(12.28)	1.15(0.68–1.93)	92(12.42)	0.82(0.54–1.24)	35(4.70)	**0.19(0.12–0.31)**
**Marital Status**											
Single	164(18.04)	24 (14.63)	1	28(17.07)	1	23(14.02)	**1**	27(16.46)	1	9(5.49)	1
Married	587(64.58)	94(16.01)	0.94(0.59–1.51)	61(10.39)	**0.62(0.39–0.98)**	62(10.56)	0.79(0.46–1.36)	68(11.58)	0.90(0.54–1.50)	43(7.33)	1.08(0.48–2.42)
Living together	49(5.39)	7 (14.29)	1.27(0.59–2.71)	3(6.12)	0.60(0.21–1.72)	5(10.20)	0.68(0.26–1.76)	7(14.29)	0.61(0.26–1.41)	4(8.16)	3.59(1.42–9.07)
Widow/separated	109(11.99)	17 (15.60)	0.78(0.38-1. 60)	17(15.6)	0.85(0.43–1.69)	17(15.6)	1.15(0.54–2.45)	17(15.6)	1.10(0.52–2.31)	15(13.76)	1.43(0.54–3.79)
**Education Status**											
None	265(29.15)	45(16.98)	1	31(11.7)	1	32(12.08)	**1**	30(11.32)	1	28(10.57)	1
Primary	169(18.59)	23(13.61)	0.84(0.52–1.36)	21(12.4)	0.81(0.49–1.33)	18(10.65)	0.75(0.43–1.31)	18(10.65)	0.91(0.52–1.58)	14(8.28)	0.75(0.42–1.35)
Middle/JHS	262(28.82)	42(16.03)	1.05(0.69–1.61)	29(11.07)	0.78(0.48–1.27)	35(13.36)	0.96(0.59–1.57)	40(15.27)	1.23(0.77–1.98)	14(5.34)	0.62(0.34–1.16)
Technical/SHS	154(16.94)	21(13.64)	0.95(0.55–1.63)	27(17.53)	1.10(0.65–1.86)	20(12.99)	0.87(0.47–1.60)	25(16.23)	1.20(0.67–2.16)	12(7.79)	0.95(0.47–1.91)
Tertiary	59(6.49)	11(18.64)	1.17(0.62–2.22)	1 (1.69)	**0.12(0.02–0.84)**	2(3.39)	0.29(0.07–1.18)	6(10.17)	0.95(0.41–2.22)	3 (5.08)	**0.27(0.08–0.92)**
**Employment**											
Ever employed	583(64.14)	48(14.72)	1	76(13.04)	1	69(11.84)	1	76(13.04)	1	54(9.26)	1
Never employed	326(35.86)	94 (16.12)	0.99(0.68–1.45)	33(10.12)	0.67(0.43–1.06)	38(11.66)	1.05(0.66–1.65)	43(13.19)	0.95 (0.61–1.46)	17(5.21)	1.03(0.58–1.84)
**District**											
Bongo	301 (33.11)	45(14.95)	1	10(3.32)	1	17(5.65)	1	29(9.63)	1	15(4.98)	1
Asunafo North	300(33.00)	36(12.00)	0.72(0.46–1.13)	16(5.33)	1.48(0.66–3.32)	36(12.00)	2.31(1.27–4.19)	62(20.67)	2.26(1.44–3.56)	18(6.00)	0.91(0.44–1.88)
Anloga	308 (33.88)	61(19.81)	1.29(0.89–1.87)	83(26.95)	**7.60(3.96–14.58)**	54(17.53)	3.32(1.93–5.71)	28(9.09)	0.96(0.57–1.60)	38(12.34)	**2.69(1.49–4.88)**

R_1_: adjusted for each socio-demographic variable in univariable analysis.

### Prevalence and detection of MNS conditions in the 3 demonstration districts

This study assessed active probable cases of MNS. The overall prevalence of any probable depression and psychotic symptoms among the study participants was 15.6% (95% CI: 13.3 – 18.1%) and 12.0% (95% CI: 10.0 − 14.3%) respectively. Prevalence of depression in men is marginally higher compared with women in the whole sample. The prevalence of probable suicidal behaviour and epilepsy was 11.8% (95% CI: 9.7 − 14.0%) and 13.1% (95% 10.9 − 15.5%) respectively. Probable AUD among the study participants was 7.8% (95% CI: 6.2 − 9.8%).

**Fig. 2:**
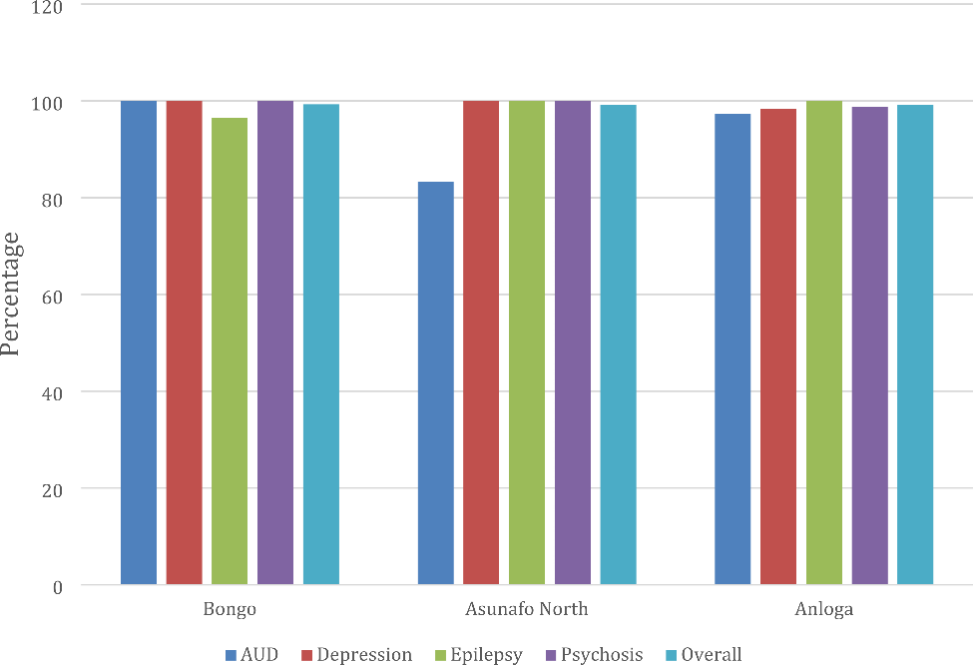
Proportion of cases of MNSconditions not detected at primary health care facilities across districts

Fig. 2 shows the proportion of patients with the priority MNS conditions (except suicide behaviour) that were missed by the health care workers across the three districts. Almost all probable cases identified by the survey team were missed (missed detection) by the health care workers with missed detection proportions ranging from 94.4–99.2% across each priority MNS condition. Missed detection for people with AUD even though very high (94.4%), was better compare with depression, psychosis and epilepsy.

Association between patient-level factors and missed detection of MNS disorders was assessed. The Fisher’s exact test shows no statistically significant association between patient level factors and case detection.

### Correlates of MNS conditions in the 3 demonstration districts

Table 2 shows patient-level correlates of the priority MNS conditions. For probable depression, independent associations of increased risk were noted only with attending a health facility in the Anloga district. We noted that the data collectors from the Anloga and Asunafo North districts included trained mental health professionals and may have accounted for the higher case detection in that district. For probable psychotic symptoms, independent associations of reduced risk were noted for being married and having a tertiary level education; all other associations were not statistically significant. Independent associations of increased risk of probable suicidal behaviour were noted for those attending a health facility in Asunafo and Anloga districts (compared to the Bongo district). For probable epilepsy, being in the age group of 35 to 44 years was associated with reduced risk of epilepsy; all other associations were not deemed statistically significant. Many more factors were associated with probable alcohol use disorder, for instance, being more than 35 years and those attending a health facility in the Anloga district had an increased risk of alcohol use disorder. Additionally, being a woman and having a tertiary education were independently associated with reduced risk of alcohol use disorder. All other associations were not statistically significant.

The study also assessed possible associations between the other MNS conditions and depression. Independent associations of increased risk were observed for having self-reported psychotic symptoms and an alcohol use disorder (RR: 1.68; 95% CI: 1.12–2.54).

## Discussion

This study set out to assess prevalence and case detection of priority MNS conditions at primary healthcare facilities in three rural settings in Ghana. Specifically we sought to estimate primary healthcare workers’ ability to detect depression, psychotic symptoms, suicidal behaviour, epilepsy, and alcohol use disorder, in comparison to detection by an independent team of researchers using screening tools for these conditions.

Prevalence of priority MNS.

An overall prevalence of probable depression among the study participants of 15.6% is lower than previous studies in Ghana that reported depression prevalence in the range of 25–62% [40, 41]. We observed a marginally higher prevalence of depression in men than in women in the whole sample. Unlike previous studies, which show a higher prevalence of depression in females, there was no significant difference in this study. The prevalence of epilepsy in this study is similar to a previous study involving five sub-Saharan Africa countries including Ghana, Kenya, South Africa, Tanzania and Uganda which reported prevalence of active epilepsy in the range of 7.8–14.8% [42, 43]. The prevalence of self-reported psychotic symptoms in the current study is similar to previous studies conducted in Ghana that reported that the prevalence of positive psychotic like experiences range from 3.8 to 41.2% [44, 45]. In other sub-Saharan African countries, a prevalence of self-reported psychotic symptoms of 3.9% and 13.9% have been reported in Tanzania [46] and Kenya [47] respectively. We note however that there are substantial methodological challenges with screening for psychosis using self-report screeners [48]. It is also possible that the self-report of psychotic-like experiences may be transient and benign, and further research would be required to ascertain specific needs for care.

The prevalence of probable alcohol use disorder of 7.8% in the current study is comparable with a previous study in Ghana among students that found alcohol use to be 6.8%, 11.1% and 12.6% for alcohol problem, lifetime drunkenness, and current alcohol use respectively [49], although a higher prevalence of 43% among the youth has been reported with more men using alcohol than women [50]. We surmise that the low prevalence of probable AUD reported in the current study is partially explained by the small number of men in this sample. Also, the prevalence of suicidal behaviour (11.8%) in the current study is comparable with a prevalence of between 5.0 and 14.8% [51] reported in a study involving five LMICs (Ethiopia, Uganda, South Africa, India and Nepal).

Detection of priority MNS conditions.

Our study reports a high rate of missed detection for MNS conditions of about 98%, indicating a low detection rate of people with MNS conditions in the routine primary healthcare facilities. The findings align with the PRIME study which reported low health worker detection of priority MNS ranging from 0 to 11.7% in five LMICs [52]. The low detection rate could be a function of the cadre of healthcare workers who attended to the routine primary healthcare attendees and who had received minimal training in mental health. In our study, participants would have been seen by either nurses/midwives who comprised of 51% of healthcare workers and/or physician assistants (48%). Participants were not seen by doctors or mental health professionals such as psychiatrists or the four main types of mental health professionals (community mental health officers, community psychiatric nurses, registered mental health nurses, and clinical psychiatric officers) within the primary healthcare system in Ghana [53]. Our findings highlight missed opportunities for mental healthcare in these primary care settings, and the need to provide training and strengthen health systems to improve detection and care for people living with MNS conditions.

Correlates of MNS conditions.

Our study found some associations between patient-level factors and the MNS conditions examined. Being married and having a tertiary level education appear to be protective against developing psychosis symptoms. These findings are similar to what has been reported from a systematic review of common mental disorders and poverty (including education and employment status) in LMICs which indicated that lower education status was positively associated with increased prevalence of common mental disorders [54]. Although some studies have reported that increasing age is associated with depression [55], Deribew et al. did not find significant association between age and depression [56].

Being a woman and having a tertiary level education were associated with reduced risk of alcohol use disorder. These findings compare with a previous study in Ghana among adults aged 15–65 years which reported that women and those with tertiary education were less likely to engage in dependent drinking [57]. Our study found an association between alcohol use disorder and depression, in keeping with previous studies which have reported a positive association between alcohol use disorder and mental illness [58]. Our study also found associations between self-reported psychotic symptoms and probable depression, which may be reflective of comorbidity between these conditions. This finding is consistent with previous studies that have reported psychiatric comorbidities among patients with schizophrenia including depression and anxiety disorders [40, 59], with depression co-occurring in persons within first episode psychosis [59].

Our study found an increased risk of the MNS conditions in some of the study districts (Asunafo North, Anloga) but not others (Bongo). This is an interesting finding with theoretical backing in terms of the exposure to place of residence and mental health outcomes. For example, the neighbourhood disorder model, the environmental stress model [60] and the social stress model [61], could explain our findings but this requires further research to explore area level factors that may explain these differences.

Implications of the survey.

The findings of the current study underscore findings from other studies in Ghana and sub-Saharan Africa [62] indicating a large treatment gap for MNS conditions in primary healthcare settings. The findings also demonstrate the need to strengthen the capacity of primary healthcare facilities to routinely detect and provide care for patients with MNS conditions. The prevalence reported in the current study may be associated with other factors that our study did not measure, such as the presence of other chronic diseases [63].

Our findings compare with previous studies that report high rates of common mental health conditions in many resource-constrained settings, indicating the need for improved detection of patients attending primary healthcare facilities [62, 64, 65]. Studies have demonstrated that developing the capacity of primary healthcare workers is a promising strategy to increasing access to mental health services and thereby addressing the missed detection rate of MNS conditions [66].

Due to the absence of specialist mental health workers and the limited mental health service provision in Ghana [67], task-sharing may be a good strategy to bolster the insufficient health workforce in an attempt to address the treatment gap and improve access to mental health service provision [68]. Nevertheless, Agyapong et al. contended that task-sharing may not be practicable in the long run given that primary healthcare workers are already over-burdened and additional responsibilities may compromise on quality health service provision [53]. Strategies to overcome this barrier may include providing ongoing training, supervision, and support; strengthening of referral pathways; improving supply of essential psychotropic medications; and inclusion of mental health indicators in routine District Health Information Management Systems (DHIMS). Another strategy to increase access to mental health services is the WHO-recommended interventions such as mhGAP-IG [6, 19] to develop the capacity of primary healthcare workers to detect mental conditions in routine primary healthcare facilities. This will help to bridge the treatment gap to achieve the universal health coverage agenda by the United Nations Sustainable Development Goal (SDG) 3 of ensuring healthy lives and promoting well-being for all at all ages [69]. It is imperative therefore, for central government and other donor organisations to provide the needed resources to support the integration and expansion of mental health services in the districts.

Strengths and limitations of the study.

A strength of this study was the adaptation of methods from previous research conducted in LMIC in the PRIME consortium [7, 70]. Nevertheless, there are some limitations. First, as this is a cross-sectional study it is not possible to infer temporal or causal relationships. Second, given not many factors were found to be associated with the prevalence of the MNS conditions examined in this study, other factors that our study did not measure could account for this. Third, the data collection was limited to fifteen health facilities within three districts across three regions of Ghana, we cannot claim that the demographic characteristics of these districts represents Ghana nationally. Therefore the prevalence reported may not be representative of the general population and should be interpreted in the context of these primary healthcare settings. Fourth, the limitations of using screening instruments for the survey need to be acknowledged. In particular, we did not conduct any clinical diagnosis by a mental health professional, limiting the conclusions that can be drawn regarding the prevalence of the MNS disorders that are reported in this study. Fifth, some of the data collectors were non-mental health professionals, even though they were trained on how to screen for probable mental health conditions. Finally, one of the data collectors from the Anloga district is a professionally trained mental health nurse, and this may have accounted for the higher prevalence reported in that district.

## Conclusions

This study found a substantial unmet need for care among people living with MNS conditions, attending primary healthcare facilities in the Bongo, Asunafo North and Anloga districts in Ghana. Despite a relatively high prevalence of probable MNS conditions, about 98% of participants who screened positive for probable depression, psychotic symptoms, epilepsy, suicidal behaviour or alcohol use disorder, were not detected by healthcare workers at the routine primary healthcare facilities. The findings demonstrate the need for improved detection and care for people living with MNS conditions in these districts. A proposed future study will evaluate changes in detection rates following implementation of district mental healthcare plans in these districts.

## Data Availability

The dataset used for the current study is available from the corresponding author on reasonable request.

## Abbreviations

AUD: Alcohol used Disorders
AUDIT: Alcohol Use Disorders Identification Test
GHS: Ghana Health Service
GHS-ERC: Ghana Health Service Ethics Review Committee
MHA: Mental Health Authority
GMHA: Ghana Mental Health Act 846
MNS: Mental, Neurological and Substance used Disorders
PHQ-9: Patient Health Questionnaire version 9
SDGs: Sustainable Development Goals
WHO: World Health Organisation
